# QRS-T Angle Predicts Cardiac Risk and Correlates With Global Longitudinal Strain in Prevalent Hemodialysis Patients

**DOI:** 10.3389/fphys.2019.00145

**Published:** 2019-02-25

**Authors:** Sofia Skampardoni, Darren Green, Katerina Hnatkova, Marek Malik, Philip A. Kalra, Dimitrios Poulikakos

**Affiliations:** ^1^Department of Renal Medicine, Salford Royal NHS Foundation Trust and The University of Manchester, Manchester, United Kingdom; ^2^National Heart and Lung Institute, Imperial College London, London, United Kingdom

**Keywords:** QRS-T angle, TCRT, sudden cardiac death, hemodialysis, cardiovascular, global longitudinal strain

## Abstract

**Background:** Cardiovascular disease is the commonest cause of death in hemodialysis (HD) patients but accurate risk prediction is lacking. The spatial QRS – T angle is a promising electrophysiological marker for sudden cardiac death risk stratification. The aim of this study was to assess the prognostic value of spatial QRS-T angle derived from standard 12 lead electrocardiograms (ECG) and its association with echocardiographic parameters in HD patients.

**Methods:** This prospective study of 178 prevalent HD patients (aged 67 ± 14 years, 72% men) collected ECG and echocardiographic data on an annual basis. Baseline echocardiograms at study entry were used for cross-sectional comparisons with ECGs. Study endpoints were all-cause mortality, cardiovascular mortality, and major adverse cardiac events (MACE). The QRS – T angle was calculated from standard 10-s ECG as the total cosine R to T (TCRT) using singular value decomposition and expressed in degrees. TCRT above 100° was defined as abnormal.

**Results:** During a follow-up period of 36 ± 19 months, 74 patients died, including 17 cardiac deaths, and 54 suffered from MACE. In multivariate Cox regression analysis, QRS-T angle by TCRT at baseline was associated with increased cardiovascular mortality both as a continuous value and dichotomized below or above 100° (HR 1.016, *p* = 0.029, CI: 1.002–1.030 and HR 3.506, CI: 1.118–10.995, *p* = 0.031 respectively) and with MACE dichotomized at 100° (HR 1.902, CI: 1.046–3.459; *p* = 0.035). In multivariate regression analysis including baseline parameters, echocardiographic global longitudinal strain (GLS) was significantly correlated with TCRT (*F* 9.648, *r*^2^ = 0.192, standardized β = 0.331, unstandardized β = 3.567, *t* = 4.4429, CI: 1.976–5.157, *p* < 0.001).

**Conclusion:** TCRT correlates with GLS and is independently associated with cardiac deaths and MACE in HD patients.

## Introduction

Cardiovascular mortality and morbidity is elevated in End Stage Renal Disease (ESRD) (Caskey et al., 2016). Renal registry data indicate that sudden death or fatal arrhythmia is the documented cause of death in approximately 26% of ESRD patients (United States Renal Data System, 2016). The pathogenesis of cardiac disease associated with sudden death in ESRD is not well understood. Traditional risk stratification models cannot be directly extrapolated to the dialysis population (Poulikakos et al., 2014) and it is speculated that the pathogenetic mechanisms differ compared to cardiac patients and to the general population (Roberts et al., 2006). The identification of high-risk patients so as to intervene to improve outcomes remains an unmet clinical need.

A recent study identified spatial QRS-T angle, an established marker of repolarization heterogeneity in cardiac patients (Zabel et al., 2000) and in the general population (Porthan et al., 2013; Waks et al., 2016), as a strong predictor of cardiovascular mortality and sudden cardiac death in incident HD patients (Tereshchenko et al., 2016). In that study (Tereshchenko et al., 2016), the calculation of the QRS-T angle was derived from signal-averaged ECG orthogonal recordings rather than resting standard 12-lead ECGs that are routinely used in clinical practice.

Amongst the existing methods of calculating the spatial QRS-T angle, (Hnatkova et al., 2017) the Total Cosine R to T (TCRT) (Acar et al., 1999) is a reliable and reproducible descriptor that can be measured from a single standard 12 lead digital ECG. In a recent pilot reproducibility study in 72 HD patients using repeated continuous intradialytic Holter ECG monitoring, abnormal TCRT was associated with major cardiac events (MACE) and mortality (Poulikakos et al., 2018).

Repolarization heterogeneity has been recently associated with structural and functional cardiac abnormalities including global longitudinal strain (GLS) derived from speckle tracking echocardiography in the general population (Biering-Sørensen et al., 2018). GLS is an emerging echocardiographic marker of uremic cardiomyopathy that predicts arrhythmic death (Hensen et al., 2018) and mortality (Waks et al., 2016) but its relationship with repolarization heterogeneity assessed by QRS-T angle has not been evaluated in HD patients.

The purpose of this study was to confirm the predictive value of TCRT calculated from standard 10-s resting ECGs and to examine the association between TCRT and various echocardiographic parameters, including GLS, in HD patients.

## Materials and Methods

### Patient Population and Protocols

Digital ECG recordings collected as part of a recently published prospective study in prevalent hemodialysis patients (Chiu et al., 2016a) that evaluated the predictive value of pulse wave velocity (PWV), left ventricular mass index (LVMI) and GLS on cardiovascular events and mortality, were available for this study. Patient characteristics, selection and recruitment process and protocols for this study have previously been described (Chiu et al., 2016a). This was a substudy of Chronic Renal Insufficiency Standards Implementation Study (CRISIS), an epidemiological study of patients with chronic kidney disease. In brief, recruited prevalent HD patients underwent sequential annual ECG recordings, echocardiography and PWV measurements and blood sampling. After phlebotomy, samples were immediately centrifuged and plasma and serum stored at -80°C. N-terminal pro-brain natriuretic peptide (BNP) and Troponin I were later quantified in batched using electrochemiluminescence (Cobas e601 automate, Roche Diagnostics Indianapolis, IN, United States).

Demographic data were extracted from medical records. Coronary artery disease (CAD) was defined as previous history of myocardial infarction and/or angina.

The study endpoints were all-cause mortality, cardiovascular mortality defined as death due to myocardial infarction, heart failure, arrhythmia or sudden cardiac death, and a composite endpoint of major cardiac events (MACE). MACE included acute coronary syndrome, coronary revascularization, hospitalization due to heart failure or arrhythmia, and sudden cardiac death. Sudden cardiac death was defined as sudden and unexpected death occurring within an hour of the onset of symptoms, or presumably due to a cardiac arrhythmia occurring in previously asymptomatic patients found dead within 24 h of last clinical contact (Al-Khatib et al., 2017). Follow up was calculated from the date of baseline ECG. All participants provided written informed consent prior to enrolment in the study. The study was approved by North West - Greater Manchester South Research Ethics Committee (15/NW/0818).

### Echocardiography and Pulse Wave Velocity

Transthoracic two-dimensional echocardiographic imaging and PWV measurements and 12-lead ECGs were obtained on single visits during a mid-week non-dialysis day. The detailed protocol of echocardiographic and PWV measurement has been described elsewhere (Chiu et al., 2016a). In brief, transthoracic echocardiography was performed using Philips Medical Systems (Philips UK Ltd., United Kingdom) with 3.5-MHz transducers. Echocardiographic measurements were in accordance with published guidance (Lang et al., 2015). The Philips Xcelera R4.1 image management system was used for offline analysis. Measurements of left ventricular volumes and left ventricular ejection fraction were performed with the biplane Simpson’s method. GLS was calculated from speckle-tracking echocardiography from apical 4- and 2-chamber views using the Philips QLAB version 9 software. Analysis was performed after the anonymization of the echocardiographic images. The GLS was calculated as average over all segmental strains of all 17 segments. M mode left ventricular (LV) mass was calculated using the Devereux formula: LV mass = 0.8 ^∗^ [1.04 ((LV internal diameter + septal wall thickness + posterior wall thickness) 3 – LV internal diameter 3) + 0.6 g]. LV mass was indexed to height 2.7 (LVMI/HT 2.7). PWV was measured with the Vicorder TM device (Skidmore Medical Ltd., Bristol, United Kingdom) using a neck and femoral cuff that were inflated to 65 mm Hg and the obtained oscillometric signal was used to calculate the pulse time delay between the two sites and consequently the aortic PWV. The mean result of two consecutive measurements was used for the analysis.

### Electrocardiographic Acquisition and Analysis

**FIGURE 1:**
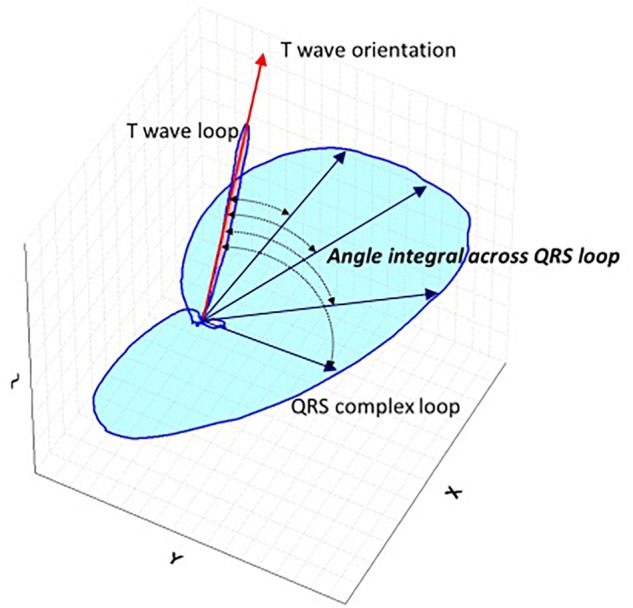
Schematic representation QRS-T angle. Schematic spatial representation of QRS and T vector loops. Main vector of T wave loop is depicted by red arrow and main vectors of QRS loop are depicted with blue straight arrows. Total Cosine R to T is the average of cosines of the angles between the three-dimensional T wave and QRS loop vectors and measures the vectorial deviation between depolarization and repolarization waves. For the three-dimensional reconstruction of the cardiac electrical signal from the surface 12 lead digital ECG, the eight independent leads are subjected to singular value decomposition to yield a system of 3 independent orthogonal leads that contain 99% of the ECG energy.

All 12-lead ECGs were recorded at a sampling rate of 500 Hz in a recumbent position and with standard lead placement (Phillips Pagewriter TC 20 electrocardiograph). Automated measurements by Phillips measurement algorithm of heart rate and ECG intervals for QRS and QT were used in the analysis. QTc was calculated using the Fridericia’s formula: QTcF = QT/^3^√RR. Software conversion routines were programmed allowing the signal to be exported from the XML Schiller format to a format suitable for further computer processing with a custom developed software. The calculation of TCRT has been previously described in detail (Acar et al., 1999). In brief, the software decomposes the ECG signal into a minimum dimensional space and reconstructs the electrical activity as a three-dimensional moving vector forming a loop during depolarization (QRS) and repolarization (T wave). The angle between the principal orientations of the QRS complex and the T wave loop (QRS –T angle) was calculated as total cosine R to T (Acar et al., 1999) and converted to degrees (Figure 1). Analysis of the digital ECG recordings was performed in a blinded manner using a purpose designed software package written in C++ and the TCRT calculations were returned to the clinical investigators for statistical analysis.

### Statistical Analysis

Analyses were performed with IBM SPSS Statistics version 22.0. Continuous variables were expressed as means ± standard deviation or as median and interquartile range if normally or non-normally distributed, respectively. *T*-test and Mann–Whitney *U* test were used for comparisons of parametric and non-parametric data as appropriate. The chi-squared test was used for categorical values where appropriate. Pearson’s correlation coefficient was used to measure the relationship between the variables and TCRT. Variables found to be significantly associated were entered into linear regression analysis to determine independent relationship with TCRT. Univariate Cox Regression analysis was used to calculate the effect of variables upon the time to outcome. TCRT and all other statistically significant variables in the univariate analysis, apart from those which correlated with TCRT in linear regression analysis, were included in multivariate Cox regression analysis to determine associations between the endpoints and variables. In the Cox regression analysis, TCRT was entered as a continuous value as well as also dichotomized at 100° based on previously published cut–off values for risk stratification for sudden cardiac death in the general population (Porthan et al., 2013). The cumulative probability of events between patient groups stratified by TCRT above and below 100° was compared by the log-rank test.

For the longitudinal analysis, the difference (Δ) between baseline TRCT and first available follow up TRCT was calculated as ΔTCRT = TCRT (follow up)-TCRT (baseline). Paired sample *t*-tests were used to compare baseline and follow up TCRT values. Analysis was performed for the total population and repeated in groups of patients with baseline TCRT below and above 100°. *P*-values < 0.05 were considered statistically significant.

## Results

### Baseline Characteristics and Associations of TCRT With Biomarkers and Echocardiographic Parameters

From 220 haemodialysis patients recruited to the original study digital ECGs were available and TCRT calculated in 179 and 98 patients at baseline and at follow up, respectively. Comparison of patient groups with TCRT above and below 100*°* showed significant differences in sex, ethnicity, diastolic BP, biplane left ventricular Ejection Fraction, GLS and LVMIht^2.7^ (Table 1). Male patients had higher baseline TCRT values than females (93 ± 41 vs. 80 ± 34°, *p* = 0.036) consistent with previous physiologic observations (Smetana et al., 2002).

**Table 1:** Baseline characteristics of the study participants according to TCRT.

	All	TCRT < 100*°*	TCRT > 100*°*	
Characteristic	(*N* = 178)	*N* = 104	*N* = 74	*P*-value
Age, years	67 ± 14	68 ± 14	66 ± 13	0.410
Men, N (%)	128 (71.9)	67 (65)	61 (83)	0.008
Diabetes Mellitus, N (%)	52 (29.2)	23 (25.8)	29 (32.6)	0.853
South Asian, N (%)	31 (17.4)	14 (13.5)	17 (21.3)	0.042
Black, N (%)	5 (2.8)	1 (1.0)	4 (4.5)	
Caucasian, N (%)	142 (79.8)	89 (85.6)	53 (74.2)	
History of Smoking	44 (24.7)	24 (23.1)	20 (27.0)	0.536
CAD, N (%)	42 (23.5)	22(21.2)	20(27)	0.363
Dialysis vintage (months)	36 ± 40	37 ± 46	34 ± 31	0.601
Heart rate, beats/min	73 ± 14	74 ± 13	74 ± 16	0.872
QRS, ms	107 ± 14	107 ± 16	108 ± 13	0.638
QTc, ms	98 ± 14	98 ± 16	406 ± 40	0.858
SBP, mmHg	141 ± 24	138 ± 20	145 ± 26	0.071
DBP, mmHg	70 ± 11	69 ± 10	72 ± 12	0.014
EDV, ml	109 ± 38	104 ± 34	117 ± 41	0.017
Biplane Ejection fraction	62 ± 13	64 ± 13	60 ± 14	0.048
LVMIht^2,7^g/m	50 ± 20	46 ± 19	56 ± 21	0.002
GLS, %	-12.9 ± 3.8	-14.3 ± 3.3	-11.2 ± 3.7	<0.0001
Troponin I ng/L	30 ± 56	28 ± 49	32 ± 70	0.670
BNP pg/mL	347 ± 520	322 ± 460	382 ± 593	0.224
PWV, m/s	8.9 ± 2.2	8.8 ± 2.0	9.0 ± 2.4	0.424

*Values are expressed as mean ± SD or N (%). BNP, N-terminal pro-brain natriuretic peptide; CAD, coronary artery disease; DBP, diastolic blood pressure; EDV, end diastolic volume; GLS, global longitudinal strain; LVMIht^2.7^, left ventricular mass indexed to Ht^2.7^; PWV, pulse wave velocity; QTc, QTc Fridericia; SBP, systolic blood pressure; TCRT, total cosine of R to T (in degrees). Statistically significant differences are highlighted with shaded areas.*

**FIGURE 2:**
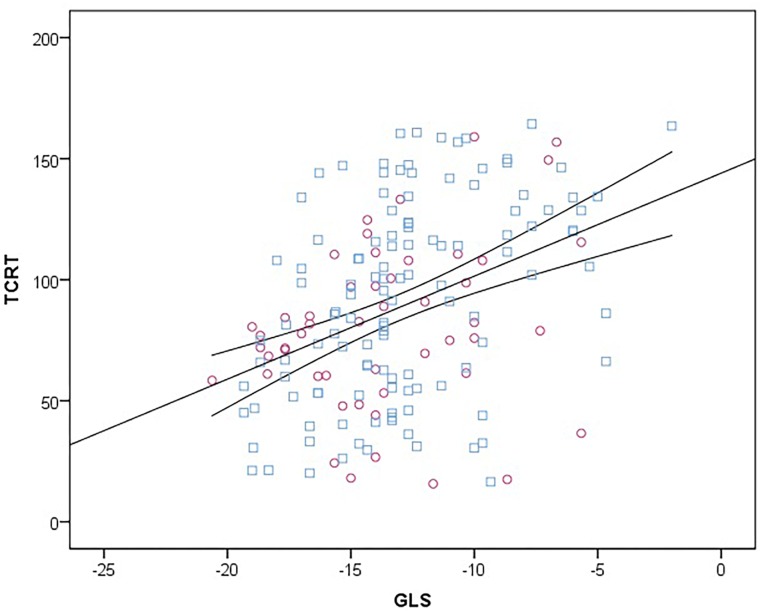
Scatter diagram between TCRT and GLS at baseline. Female subjects are represented with purple circles and male subjects with blue squares. Linear regression line (straight) is plotted with 95% confidence intervals (curved lines). TCRT, total cosine R to T; GLS, global longitudinal strain.

On univariate analysis TCRT correlated with GLS (*r* = 0.398, *p* < 0.001), LVMIht^2.7^ (*r* = 0.212, *p* = 0.005), systolic BP (*r* = 0.155, *p* = 0.043) and diastolic BP (*r* = 0.172, *p* = 0.024). On multivariate linear regression analysis including LVMIht^2.7^, systolic BP, diastolic BP and GLS, only GLS remained significantly correlated with TCRT in the model explaining 19% of the variance (*F* 9.648, *r*^2^ = 0.192, standardized β = 0.331, unstandardized β = 3.567, *t* = 4.4429, CI: 1.976–5.157, *p* < 0.001). The relationship between GLS and TCRT is shown in Figure 2.

### Outcomes

During a mean follow up period of 36 ± 19 months, there were 74 deaths including 17 cardiovascular deaths (9 due to myocardial infarction, 7 were sudden cardiac deaths and 1 was secondary to heart failure), 54 MACE and 32 subjects received a kidney transplant. TCRT values according to outcomes are presented in Table 2.

**Table 2:** TCRT according to end points and to transplant status during follow up.

Outcomes		TCRT	*P*-value
All Cause Deaths, N	Yes (74)	92 ± 38	0.366
	No (104)	87 ± 40	
Cardiac Deaths, N	Yes (17)	114 ± 37	0.006
	No (161)	87 ± 39	
MACE	Yes (54)	98 ± 39	0.043
	No (124)	85 ± 39	
Transplanted	Yes (32)	86 ± 36	0.634
	No (146)	89 ± 40	

*Values are expressed as mean ± SD or N. MACE: major cardiac events, TCRT, total cosine of R to T (in degrees). Statistically significant differences are highlighted with shaded areas.*

### All-Cause Mortality

On univariate Cox regression analysis, the factors associated with increased all-cause mortality were: age, history of CAD, QRS duration, QTc duration, End Diastolic Volume, BNP, Systolic Blood Pressure and PWV. TCRT was found not to have a statistically significant association with all-cause mortality. On multivariate analysis, only age and PWV maintained an association with all-cause mortality. Results of univariate and multivariate Cox regression analysis for all-cause mortality are presented in Table 3.

**Table 3:** Variables associated with increased all-cause mortality by univariate and multivariate Cox regression analysis.

Variables	All-cause Mortality (*N* = 74)	
	HR (95% CI)	*P*-value
	*Univariate Cox Regression Analysis*	
Age (years)	1.034 (1.014–1.054)	0.001
Diabetes Mellitus	1.650 (1.034–2.634)	0.036
Prevalent CAD	1.679 (1.042–2.707)	0.033
QRS duration	1.017 (1.004–1.031)	0.010
QTc duration	1.017 (1.001–1.034)	0.033
End Diastolic Volume	1.008 (1.002–1.014)	0.010
BNP	1.001 (1.000–1.001)	0.005
Systolic BP (mmHg)	1.010 (1.001–1.019)	0.027
PWV	1.227 (1.128–1.333)	<0.001
	*Multivariate Cox Regression Analysis*	
Age (years)	1.038 (1.016–1.059)	0.01
PWV	1.317 (1.180–1.470)	<0.001

*BNP, N-terminal pro-brain natriuretic peptide; BP, blood pressure; CAD, coronary artery disease; CI, confidence interval; PWV, pulse wave velocity; N, number of patients.*

### Cardiovascular Mortality

**FIGURE 3:**
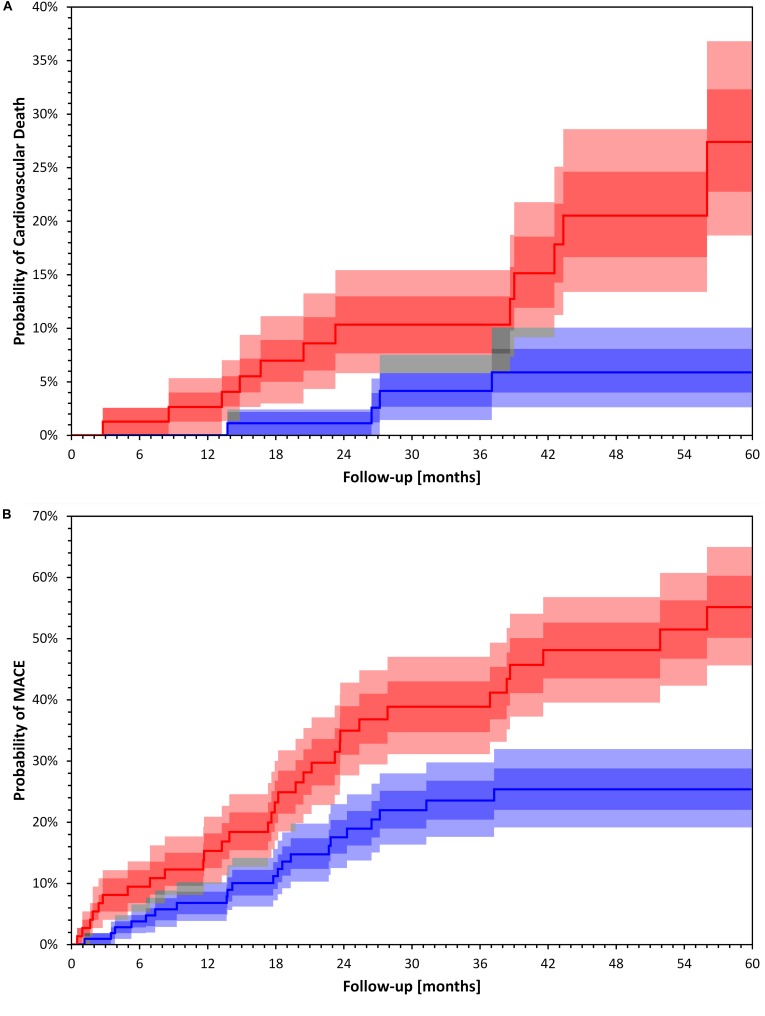
Kaplan Meier event probability curves for Cardiac Death **(A)** and Major Cardiac Events **(B)**. Kaplan Meier event probability curves for Cardiac Death **(A)** log rank p = 0.004 and Major Cardiac Events **(B)** log rank p = 0.002 for patient groups stratified by a TCRT above and below 100°. Bands represent interquartile ranges (IQR) (darker colors) and 80% percentiles (lighter colors). The gray areas show the overlaps. The dark, middle and light gray correspond to overlaps of IQRs, IQR+80% and 80%+80%, respectively. The bands have been calculated with bootstrap with 10000 random repetitions.

On univariate analysis, variables associated with cardiovascular deaths were TCRT used as a continuous variable as well as dichotomized at 100*°*, GLS, Troponin I and history of CAD. Patients with TCRT greater than 100° had higher cardiovascular mortality (*p* = 0.004 by log-rank, Figure 3A). In multivariate analysis GLS was not included in the same model with TCRT because GLS and TCRT were correlated with each other. On multivariate analysis only TCRT remained as a significant predictor both as a continuous value and when greater than 100°. Multivariate analysis including GLS and excluding TCRT did not reveal any significant associations. Results of univariate and multivariate Cox regression analysis for cardiovascular mortality are presented in Table 4.

**Table 4:** Variables associated with increased cardiovascular mortality by univariate and multivariate Cox regression analysis.

Variables		Cardiovascular Mortality (*N* = 17)	
		HR (95% CI)	*P*-value
		*Univariate Cox Regression Analysis*	
TCRT	Continuous	1.018 (1.004–1.031)	0.010
	>100°	4.443 (1.447–13.638)	0.009
GLS		1.144 (1.012–1.293)	0.031
Troponin I		1.005 (1.000–1.010)	0.046
Prevalent CAD		2.755 (1.063–7.145)	0.037
		*Multivariate Cox Regression Analysis^∗^*	
TCRT	Continuous	1.016 (1.002–1.030)	0.029
	>100°	3.506 (1.118–10.995)	0.031

*CAD, coronary artery disease; CI, confidence interval; GLS, global longitudinal strain; N, number of patients; TCRT, total cosine R to T expressed in degrees.*

*^∗^GLS was not included in the multivariate model together with TCRT due to autocorrelation.*

### Major Cardiac Events

On univariate Cox regression analysis, the factors associated with MACE were TCRT used as a continuous variable and dichotomized at 100*°*, PWV, GLS, BNP, and history of CAD. Patients with TCRT greater than 100*°* had higher cumulative probability of MACE (*p* = 0.002 by log-rank, Figure 3B). Because of the significant association between TCRT and GLS we did not include GLS and TCRT in the multivariate model. Using multivariate Cox regression analysis TCRT dichotomized at 100° and PWV remained independent predictors of MACE. TCRT as a continuous variable did not remain significant in the multivariate analysis. Results of univariate and multivariate Cox regression analysis for MACE are presented in Table 5. On multivariate analysis including GLS and excluding TCRT, PWV, and CAD remained significant but not GLS.

**Table 5:** Variables associated with major cardiac events (MACE) by univariate and multivariate Cox regression analysis.

Variables		MACE (*N* = 54)	
		HR (95% CI)	P value
		*Univariate Cox Regression Analysis*	
TCRT	Continuous	1.007 (1.000–1.014)	0.048
	>100°	2.322 (1.328–4.061)	0.008
PWV		1.133 (1.019–1.260)	0.021
GLS		1.115 (1.040–1.196)	0.002
BNP		1.001 (1.000–1.001)	0.011
Prevalent CAD		2.245 (1.280–3.939)	0.005
		*Multivariate Cox Regression Analysis model 1 ^1^*	
TCRT > 100°		1.902 (1.046–3.459)	0.035
PWV		1.141 (1.021–1.276)	0.020
		*Multivariate Cox Regression Analysis model 2 ^2^*	
PWV		1.159 (1.028–1.306)	0.016
Prevalent CAD		1.955 (1.023–1.306)	0.043

*BNP, N-terminal pro-brain natriuretic peptide; CAD, history of coronary artery disease; CI, confidence interval; GLS, global longitudinal strain; PWV, pulse wave velocity; HR, hazard ratio; TCRT, total cosine of R to T dichotomized at 100° ^1^Multivariate Cox regression model not including GLS. ^2^Multivariate Cox regression model not including TCRT.*

### Longitudinal Changes in TCRT

There were 98 analyzable follow up ECGs. Median time interval from baseline to follow up ECG was 13 (IQR 7) months. There were 31 MACE events, 9 cardiovascular deaths and 40 all-cause deaths in this patient sub-group. From the 31 MACE events, 20 MACE events occurred after the follow up ECG. TCRT increased from baseline to follow up (86 ± 41° vs. 94 ± 41°, *p* = 0.016). There was no significant increase in TCRT at follow up in the group with baseline TCRT > 100° (128 ± 17° vs. 127 ± 27°, *p* = 0.794) as opposed to the group with baseline TCRT < 100° (58 ± 24° vs. 72 ± 34°, *p* = 0.004). Longitudinal changes of TCRT according to baseline values dichotomized at 100° are presented in Table 6.

**Table 6:** Longitudinal changes of TCRT according to baseline values dichotomized at 100°.

Variable	Total *N* = 98	Baseline TCRT < 100° *N* = 59			Baseline TCRT > 100° *N* = 39
Baseline TCRT	86 ± 41	58 ± 24			128 ± 17
Follow up TCRT	94 ± 41	72 ± 34			127 ± 27
Follow up TCRT > 100° N(%)		7(12)			34(87)
DTCRT	3.41 (32.91)	7.60(35.10)			0.97(23.18)
Transplanted N (%)	9 (9)	7 (12)			2 (5)

		**TCRT progressed > 100°**			
		**Yes (*N* = 7)**	**No (*N* = 52)**	***p*-value**	

MACE, N%	31 (32)	4 (57)	15 (29)	0.048	17 (44)
Cardiac Death N%	9 (9)	1 (14)	6 (11)	0.225	7 (18)
Mortality N%	40 (41)	1 (14)	17 (32)	0.011	17 (44)

*TCRT values are expressed as mean ± SD, DTCRT: TCRT (follow up)-TCRT (baseline), Outcomes are presented as number N (%). MACE, major cardiac events; TCRT, total cosine of R to T.*

## Discussion

In our cohort of prevalent HD patients, QRS – T angle calculated as TCRT from standard surface 12 lead ECGs, carried independent prognostic value for major adverse cardiac events and cardiac mortality. We also observed an association between TCRT and GLS which is a recognized descriptor of uremic cardiomyopathy (Kramann et al., 2014).

### QRS-T Angle

Our results, in line with three previously published studies in HD patients (de Bie et al., 2013b; Tereshchenko et al., 2016; Poulikakos et al., 2018) confirm the strong predictive value of the spatial QRS-T angle for cardiac outcomes in HD patients. Tereshchenko et al. (2016) measured the QRS-T angle from signal averaged ECGs in a large cohort of incident HD patients, calculated as the angle between the mean QRS vector and the peak T vector, and identified optimal cut off prognostic value with normal QRS – T angle of 75° for all-cause mortality, cardiovascular mortality and sudden cardiac death. de Bie et al. (2013b) analyzed standard 10 s ECGs in incident HD patients defining the QRS – T angle as the angle between the mean vectors of the QRS and T and used a cut off value of 130° in males and 116° in females for all-cause mortality and sudden cardiac death. The discrepancies between the cut off values quoted in different studies may be explained by the differences in the methods and types of ECG recordings used.

In our study, we used TCRT (total cosine of R to T) as an expression of the QRS – T angle. In a recent comparative study between different methods of calculation of the QRS-T angle in cardiac patients and healthy individuals, TCRT was found to be more reliable for risk prediction in cardiac patients compared to other methods for QRS –T angle calculation (Hnatkova et al., 2017). It has been postulated that in the presence of cardiac abnormalities the complexity of the vectorocardiographic loops increases and their spatial orientation is less clearly determined. Therefore, the TCRT technology, by integrating all possible directions of the vectrocardiographic loops as average cosine of all angles, is likely to reflect more accurately the repolarization heterogeneity aberration and improve the risk assessment.

During follow up, TCRT increased in those patients with baseline values below 100°. Large studies in the general population have reported that abnormal increases (Biering-Sørensen et al., 2018) or rapid worsening of QRS-T angle (Biering-Sørensen et al., 2018) is associated with increased risk of sudden cardiac death. The small number of MACE events in the patients with available follow up ECGs, and the relative large proportion of MACE events in the time interval between the ECGs that may have affected the second ECG, prevented us from analyzing the impact of longitudinal changes on MACE and cardiovascular death in this study. Future prospective studies should examine the potential utility of serial assessments of TCRT to characterize the evolution of uremic cardiomyopathy and identify the transition to high risk profiles.

In contrast to the two previous studies in HD patients (de Bie et al., 2013b; Tereshchenko et al., 2016) we did not find an association between higher values of TCRT and all-cause mortality in this cohort of patients. However the aforementioned studies (de Bie et al., 2013b; Tereshchenko et al., 2016) were performed in incident dialysis patients and these patients are known to suffer from particularly high mortality rates during the first 3 months after dialysis initiation (Poulikakos et al., 2014). The third study (Poulikakos et al., 2018) applied intradialytic ECG recordings during the HD sessions of prevalent HD patients and was therefore able to recruit patients with multiple comorbidities and higher mortality risk who would otherwise find it difficult to participate if they had to attend on a non-dialysis day for study screening. We previously reported that patients recruited to our study had longer survival compared to non-participants (Chiu et al., 2016b); patients that denied consent had an adjusted hazard ratio of 1.70, 95% CI 1.10–2.52 and therefore it is possible that recruitment bias may have affected the overall survival outcomes. Furthermore, our prevalent dialysis patients were followed up annually with detailed cardiovascular and clinical assessment and it is possible that such close cardiovascular monitoring enabled early identification and management of cardiovascular disease.

### Repolarization Heterogeneity and Cardiac Function

Two previous studies have explored the association between echocardiographic abnormalities and QRS-T angle in dialysis patients. In the study by Tereshchenko et al. (2016) increased QRS-T angle was associated with left ventricular hypertrophy but not with left ventricular ejection fraction. In another study of 94 HD patients (de Bie et al., 2013a) left ventricular ejection fraction and LV dysynchrony were indendent predictors of abnormal QRS-T angle (de Bie et al., 2013a). Speckle tracking echocardiography was not included in either study. To our knowledge, the association between TCRT and GLS in a HD population has not been previously reported. Our results are in line with recent large study in the general population (Biering-Sørensen et al., 2018), showing that the QRS-T angle and other measures of repolarization heterogeneity are associated with subclinical abnormalities in cardiac structure and function. In the study by Biering-Sørensen et al. (2018) QRS-T angle explained 10-20% of the variance of GLS depending on the linear regression model used which is similar to the modest 19% of variance in GLS explained by TCRT in our study. The association between repolarization heterogeneity and cardiac function that we observe in HD patients may represent the epiphenomenon of underlying myocardial fibrosis leading to both electrical and functional aberrations or it may reflect the effect of repolarization heterogeneity on cardiac function via inducing heterogeneity and dysynchrony in mechanical contraction within the different myocardial layers. An association between repolarization heterogeneity and mechanical contraction assessed by GLS, in the absence of underlying myocardial fibrosis, has been shown in observational studies in subjects with long QT syndrome (Haugaa et al., 2010; Leren et al., 2015), a condition that is characterized by loss of function potassium channel mutations leading to prolonged action potential duration (APD). In experimental rat models of chronic kidney disease that used optical mapping techniques, increase in APD and lengthening of pacing cycle thresholds to induce APD alterans were detected in CKD rats compared to controls CKD rats; ventricular fibrillation was induced in 9/12 of CKD rats compared to 2/9 normal rats indicating heightened risk of ventricular arrhythmias (Hsueh et al., 2014). Interestingly, although selected markers of myocardial fibrosis, such as mRNA of TGF-b and micro RNA 21, were upregulated in CKD-rats compared to control rats there was no statistically significant difference in myocardial fibrosis between the two groups. The level of fibrosis observed in diseased animals using this rat model of CKD (rats with autosomal dominant polycystic kidney disease) was lower than previous studies using 5/6 nephrectomy model CKD rats (Koleganova et al., 2009; Kramann et al., 2014). In this animal model the increased vulnerability to arrhythmias preceded the development of fibrosis and this finding supports a causative link between altered electrophysiological properties and malignant arrhythmias independent of myocardial fibrosis. However, the effects of electrophysiological aberrations upon the myocardial mechanics were not assessed in this study.

The potential impact of repolarization heterogeneity on cardiac function is also supported by the observation that changes in repolarization heterogeneity measured by 5 ECG parameters including QRS-T angle typically preceded echocardiographic abnormalities in a recently published large general population study (Biering-Sørensen et al., 2018).

The potential relative contribution of repolarization heterogeneity on cardiac function and its relationship with underlying myocardial fibrosis cannot be established from our observational study. Future research to elucidate the link between repolarization heterogeneity and mechanical dysfunction may have important implications in understanding the pathophysiology of uremic cardiomyopathy and in optimizing risk assessment in HD patients. The QRS-T angle assessed by TCRT and GLS, although interrelated, carry prognostic information that is independent of each other and which should be assessed in combination in risk assessment models.

### Limitations

The limitations of this study including the relatively small sized population and recruitment selection bias leading to lower mortality have previously been described (Chiu et al., 2016a,b). Due to the small number of arrhythmic deaths, we did not have the statistical power to investigate associations of TCRT with sudden cardiac death and cardiac arrest separately. Also, we did not manage to investigate follow up ECGs in a large number of patients for the longitudinal ECG analysis thus limiting the scope of the statistical analysis for the longitudinal changes.

## Conclusion

In conclusion, our study has demonstrated that QRS-T angle calculated by TCRT, an electrocardiographic marker of repolarization heterogeneity that can be derived from standard 10 s ECGs, carries independent prognostic significance for cardiac mortality risk in our HD cohort and it is correlated with GLS. Further prospective studies with longitudinal cardiovascular monitoring including a combination of electrophysiologic and cardiac structural and functional assessment from patients with earlier stages of chronic kidney disease are required to characterize the determinants of the progression of uremic cardiomyopathy and identify potential opportunities for interventions. In light of the consistently strong risk prediction signal of the QRS-T angle in the renal population we suggest that standardization of the assessment of QRS-T angle is important to enable identification of cut-off points indicating high risk to be used in routine clinical practice.

## Author Contributions

SS and DG collected, analyzed, and interpreted the data, drafted the article, and contributed to statistical expertise. KH processed ECG signal and drafted the article. MM conceived and designed the study, processed the ECG signal, interpreted the data, and critically revised the article. PK conceived and designed the study, interpreted the data, and critically revised the article. DP conceived and designed the work, analyzed and interpreted the data, contributed to statistical expertise, and critically revised the article.

## Conflict of Interest Statement

The authors declare that the research was conducted in the absence of any commercial or financial relationships that could be construed as a potential conflict of interest.

## References

[B1] Acar B. Yi G. Hnatkova K. Malik M. (1999). Spatial, temporal and wavefront direction characteristics of 12-lead T-wave morphology. *Med. Biol. Eng. Comput.* 37 574–584. 10.1007/BF02513351 10723894

[B2] Al-Khatib S. M. Stevenson W. G. Ackerman M. J. (2017). AHA/ACC/HRS guideline for management of patients with ventricular arrhythmias and the prevention of sudden cardiac death: executive summary: a report of the american college of cardiology/american heart association task force on clinical practice guidelines and the heart rhythm society. *Heart Rhythm* 15 e190–e252. 10.1016/j.hrthm.2017.10.035 29097320

[B3] Biering-Sørensen T. Kabir M. Waks J. W. (2018). Global ECG measures and cardiac structure and function: the aric study (atherosclerosis risk in communities). *Circ. Arrhythm Electrophysiol.* 11:e005961. 10.1161/CIRCEP.117.005961 29496680 PMC5836803

[B4] Caskey F. Castledine C. Dawnay A. Farrington K. Fogarty D. Fraser S. (2016). *18th Annual Report of the Renal Association, UK Renal Registry*. Available at: https://www.renalreg.org/wp-content/uploads/2017/09/19th-Annual-Report_web_book.pdf

[B5] Chiu D. Abidin N. Johnstone L. (2016a). Novel approach to cardiovascular outcome prediction in haemodialysis patients. *Am. J. Nephrol.* 43 143–152. 10.1159/000444924 27064437

[B6] Chiu D. Green D. Abidin N. (2016b). Non-recruitment to and selection bias in studies using echocardiography in hemodialysis patients. *Nephrology* 22 864–871. 10.1111/nep.12865 27470704

[B7] de Bie M. K. Ajmone Marsan N. Gaasbeek A. (2013a). Echocardiographical determinants of an abnormal spatial QRS-T angle in chronic dialysis patients. *Nephrol. Dial. Transplant.* 28 3045–3052. 10.1093/ndt/gft347 24092849

[B8] de Bie M. K. Koopman M. G. Gaasbeek A. (2013b). Incremental prognostic value of an abnormal baseline spatial QRS-T angle in chronic dialysis patients. *Europace* 15 290–296. 10.1093/europace/eus306 23024335

[B9] Haugaa K. H. Amlie J. P. Berge K. E. Leren T. P. Smiseth O. A. Edvardsen T. (2010). Transmural differences in myocardial contraction in long-QT syndrome: mechanical consequences of ion channel dysfunction. *Circulation* 122 1355–1363. 10.1161/CIRCULATIONAHA.110.960377 20855658

[B10] Hensen L. C. R. Goossens K. Podlesnikar T. (2018). Left ventricular mechanical dispersion and global longitudinal strain and ventricular arrhythmias in predialysis and dialysis patients. *J. Am. Soc. Echocardiogr.* 31 777–783. 10.1016/j.echo.2018.01.010 29534843

[B11] Hnatkova K. Seegers J. Barthel P. (2017). Clinical value of different QRS-T angle expressions. *Europace* 20 1352–1361. 10.1093/europace/eux246 29016907 PMC6075511

[B12] Hsueh C. H. Chen N. X. Lin S. F. (2014). Pathogenesis of arrhythmias in a model of CKD. *J. Am. Soc. Nephrol.* 25 2812–2821. 10.1681/ASN.2013121343 24854269 PMC4243359

[B13] Koleganova N. Piecha G. Ritz E. Gross M. L. (2009). Calcitriol ameliorates capillary deficit and fibrosis of the heart in subtotally nephrectomized rats. *Nephrol. Dial. Transplant.* 24 778–787. 10.1093/ndt/gfn549 18829613

[B14] Kramann R. Erpenbeck J. Schneider R. K. (2014). Speckle tracking echocardiography detects uremic cardiomyopathy early and predicts cardiovascular mortality in ESRD. *J. Am. Soc. Nephrol.* 25 2351–2365. 10.1681/ASN.2013070734 24700873 PMC4178432

[B15] Lang R. M. Badano L. P. Mor-Avi V. (2015). Recommendations for cardiac chamber quantification by echocardiography in adults: an update from the american society of echocardiography and the european association of cardiovascular imaging. *Eur. Heart J. Cardiovasc. Imag.* 16 233–270. 10.1093/ehjci/jev014 25712077

[B16] Leren I. S. Hasselberg N. E. Saberniak J. (2015). Cardiac mechanical alterations and genotype specific differences in subjects with long QT syndrome. *JACC Cardiovasc. Imag.* 8 501–510. 10.1016/j.jcmg.2014.12.023 25890583

[B17] Porthan K. Viitasalo M. Toivonen L. Havulina A. S. Jula A. Tikkanen J. T. (2013). Predictive value of electrocardiographic T-wave morphology parameters and T-wave peak to T-wave end interval for sudden cardiac death in the general population. *Circ. Arrhythm Electrophysiol.* 6 690–696. 10.1161/CIRCEP.113.000356 23881778

[B18] Poulikakos D. Banerjee D. Malik M. (2014). Risk of sudden cardiac death in chronic kidney disease. *J. Cardiovasc. Electrophysiol.* 25 222–231. 10.1111/jce.12328 24256575

[B19] Poulikakos D. Hnatkova K. Banerjee D. Malik M. (2018). Association of QRS-T angle and heart rate variability with major cardiac events and mortality in hemodialysis patients. *Ann. Noninvasive Electrocardiol.* 23:e12570. 10.1111/anec.12570 29938866 PMC6931824

[B20] Roberts M. A. Hare D. L. Ratnaike S. Ierino F. L. (2006). Cardiovascular biomarkers in CKD: pathophysiology and implications for clinical management of cardiac disease. *Am. J. Kidney Dis.* 48 341–360. 10.1053/j.ajkd.2006.06.005 16931208

[B21] Smetana P. Batchvarov V. N. Hnatkova K. Camm A. J. Malik M. (2002). Sex differences in repolarization homogeneity and its circadian pattern. *Am. J. Physiol. Heart Circ. Physiol.* 282 H1889–H1897. 10.1152/ajpheart.00962.2001 11959656

[B22] Tereshchenko L. G. Kim E. D. Oehler A. (2016). Electrophysiologic substrate and risk of mortality in incident hemodialysis. *J. Am. Soc. Nephrol.* 27 3413–3420. 10.1681/ASN.2015080916 27129390 PMC5084888

[B23] United States Renal Data System (2016). *USRDS Annual Data Report: Epidemiology of Kidney Disease in the United States*. Bethesda, MD: National Institutes of Health.

[B24] Waks J. W. Sitlani C. M. Soliman E. Z. Kabir M. Ghafoori E. Biggs M. L. (2016). Global electric heterogeneity risk score for prediction of sudden cardiac death in the general population: the atherosclerosis risk in communities (ARIC) and cardiovascular health (CHS) studies. *Circulation* 133 2222–2234. 10.1161/CIRCULATIONAHA.116.021306 27081116 PMC4899170

[B25] Zabel M. Acar B. Klingenheben T. Franz M. R. Hohnloser S. H. Malik M. (2000). Analysis of 12-lead T-wave morphology for risk stratification after myocardial infarction. *Circulation* 102 1252–1257. 10.1161/01.CIR.102.11.1252 10982539

